# A Novel UV Barrier Poly(lactic acid)/Poly(butylene succinate) Composite Biodegradable Film Enhanced by Cellulose Extracted from Coconut Shell

**DOI:** 10.3390/polym15143000

**Published:** 2023-07-10

**Authors:** Xiaoyan He, Lisheng Tang, Jun Zheng, Yuanyuan Jin, Ruobin Chang, Xiaoquan Yu, Yihu Song, Ran Huang

**Affiliations:** 1 Department of Polymer Science and Engineering, Zhejiang University, Hangzhou 310027, China; 2 Department of Material Science and Engineering, Research Institute of Zhejiang University-Taizhou, Taizhou 318000, China; 3 Academy for Engineering and Applied Technology, Fudan University, Shanghai 200433, China

**Keywords:** coconut shell extract, cellulose, biodegradable films, UV barrier property

## Abstract

Cellulose was extracted from coconut shell powder (CSP) as a renewable biomass resource and utilized as a reinforcing material in poly(lactic acid)/poly(butylene succinate) (PLA/PBS) solvent casting films. The extraction process involved delignification and mercerization of CSP. Microscopic investigation of the extracted microfibers demonstrated a reduction in diameter and a rougher surface characteristic compared to the raw CSP. The cellulose prepared in this study exhibited improved thermal stability and higher crystallinity (54.3%) compared to CSP. The morphology of the cycrofractured surface, thermal analysis, mechanical property, and UV transmittance of films were measured and compared. Agglomeration of 3 wt.% of cellulose was observed in PLA/PBS films. The presence of cellulose higher than 1 wt.% in the PLA/PBS decreased the onset decomposition temperature and maximum decomposition temperature of films. However, the films loading 3 wt.% of cellulose had a higher char formation (5.47%) compared to neat PLA/PBS films. The presence of cellulose promoted the formation of non-uniform crystals, while cellulose had a slightly negative impact on crystallinity due to the disruption of polymer chains at lower cellulose content (0.3, 0.5 wt.%). The mechanical strength of PLA/PBS films decreased as the cellulose content increased. Moreover, PLA/PBS film with 3 wt.% of cellulose appeared to show a 3% and 7.5% decrease in transmittance in UVC (275 nm) and UVA (335 nm) regions compared to neat PLA/PBS films while maintaining a certain transparency.

## 1. Introduction

With the rapid depletion of natural resources and the escalating environmental issues caused by the recalcitrant wastes of non-biodegradable plastics, there is a massive demand for the utilization of biodegradable and renewable raw materials [1]. Polylactic acid (PLA), as a biodegradable aliphatic polyester, has received extensive attention due to its reliable industrial-scaled productions and excellent properties, especially its processability and wide range of applications in sustainable and environmentally friendly products [2,3]. Although the many merits of PLA guarantee its utilization in various fields such as biological engineering, daily tableware and containers, agriculture, and automotive [4,5,6], the application of PLA in the biocomposite packaging field is still limited by its brittleness, poor thermal stability, low impact strength, low flexibility, and slow crystallization [7,8,9,10]. Many approaches have been explored to further remedy the limitations mentioned above, for example, grafting with suitable polymers via melt compounding or solvent casting, or physical blending with plasticizers [11,12]. It is confirmed that blending PLA with other biodegradable polymers with low glass transition temperatures (T_g_), such as poly(butylene succinate) (PBS) (T_g_ ≈ −32 °C) [13,14,15,16,17], poly(butylene succinate-co-butylene adipate) (PBSA) (T_g_ ≈ −45 °C) [18,19,20], and poly(butylene adipate-co-terephthalate) (PBAT) (T_g_ ≈ −50 °C), is an efficient solution since it not only maintains the biodegradability but also well tunes the T_g_, addressing the brittleness problem [19,21,22,23,24].

However, it is important to note that blending PBS with PLA can result in a loss of clarity, especially in applications where transparency is necessary. The phase separation in the PLA/PBS blend tends to make it translucent. Achieving transparency is not solely dependent on miscibility but also relies on factors such as crystallite size and the balance between amorphous and crystalline phases to minimize light scattering. Wang et al. demonstrated that incorporating DCP (0.05–0.2 phr) could enhance the percent transmittance of the PLA/PBS (80/20 wt.%) blend [25]. This improvement could be attributed to increased compatibility, a reduction in the size of the PBS domains, and a decrease in PBS crystallinity. In addition, the random copolymer of PLA and PBS (rPBSL) also enhanced the film clarity as a consequence of the compatibility of PLA/PBS (80/20) blend as well as the decrease in the size of spherulite diameter (~15 mm) [12].

On the other hand, one principal drawback of PBS is the substantial increase in cost. Therefore, achieving a balance between the strength, toughness, and price of PLA composites requires a synergistic approach that incorporates low-cost and easily obtainable fillers. From this viewpoint, modification of PLA/PBS by physical blending and loading of a filler is an economic and effective approach to overcome these limitations.

Lignocellulosic fibers obtained from plant and cellulose-based sources are common bio-fillers for reinforcing polymer matrices [26,27,28]. Notably, quality fibrous fillers can be obtained from agricultural wastes such as bagasse, wheat straws, rice husks, groundnut shells, coconut husks, and cotton stalks [29]. Coconut shells are primarily composed of cellulose, hemicellulose, and lignin, sharing a chemical composition that closely resembles wood and is suitable for value-added extraction [30]. In China, the annual production of coconut shell residues exceeds 400 million tons, representing immense potential for conversion into fuels and chemicals. In this context, the fractionation of a coconut shell material in its main constituents—cellulose, hemicellulose, and lignin—is of great importance for the development of a sustainable economy [31]. Cellulose extraction is commonly achieved through sulfuric acid or nitric acid hydrolysis. However, the resulting cellulose products usually have limited thermal stability, indicated by a maximum degradation temperature (T_max_) of 250 °C, which would limit utilization in various melt processing techniques like injection molding, twin-screw compounding, and extrusion [32]. Alternative procedures for the extraction of cellulose have been proposed and successfully implemented; mild acids, such as glacial acetic acid, along with sodium chlorite, have been employed under ultrasonic or microwave irradiation. These alternative methods aim to enhance the extraction process while maintaining or improving the thermal stability of the extracted cellulose, enabling its application in a broader range [33,34,35]. Cotton linters were partially hydrolyzed in dilute acid. These facts suggest that amorphous cellulose in the bulk is not accessible for hydrolysis and that microfibril bundles are hydrolyzed through a surface reaction process [36].

As mentioned above, PLA, PBS, and cellulose are some of the most promising candidates and play vital roles in contributing toward the marketing of bioplastics designed for sustainable packaging. None of them alone can meet the demands for various structural materials in packaging applications. For food packaging applications, a barrier against UV light is essential to inhibit the decomposition of packed foodstuffs [37]. PLA/3.0%Napier cellulose nanowhiskers (NWCs) film exhibited UVA and UVB transmittance of 7.49% and 4.02%, respectively, making it suitable for packaging materials [38].

To the best of our knowledge, there has been no report on PLA/PBS composite films reinforced by cellulose extracted from coconut shells. Herein, this work investigated the detailed cellulosic preparations from coconut shells by yield, thermal stability, crystallinity, spectroscopic characterization, and morphology and aims at exploiting the potential of the cellulose extracted from coconut shells to reinforce PLA/PBS films. The thermal stability, crystallization behavior, UV-Vis transmittance, and cryofractured surface characteristics of the films were investigated, revealing features that make the films promising for applications as environmentally friendly food packaging products. 

## 2. Materials and Methods

### 2.1. Materials

Polylactic acid (PLA) FY801 (Mw = 120,000 g/mol, stereochemical purity in L-isomer 99.8%, MFR 4.1 g/10 min at 190 °C) was purchased from Anhui BBCA Biochemical Futerro PLA Co., Ltd., Bengbu, China. Poly (butylene succinate) (PBS) TH803S (Mw = 140,000 g/mol, MFR 7.5 g/10 min at 190 °C) was provided by Xinjiang Blue Ridge Tunhe Chemical Industry Co., Ltd., Changji, China. Sodium chlorite (purity 80%), glacial acetic acid (purity 99.7%), sodium hydroxide (NaOH, purity 97%), dichloromethane (DCM, purity 99.9%), and all other chemicals were supplied by Macklin Chemical Co., Ltd., Shanghai, China. The coconuts were kindly donated by Zhejiang Freenow Food Co., Ltd., Jiaxing, China. Before use, the white portion of the coconut shells was peeled off, oven-dried, and ground to pass through a 40-mesh sieve.

### 2.2. Extraction of Cellulose from Coconut Shells

A total of 12 g of CSP was sequentially treated with 390 mL of distilled water, 3 mL of glacial acetic acid, and 4.5 g of sodium chlorite. The reactants were continuously stirred for 1 h in a thermostatic water bath at 75 °C [33]. After 1 h, another 3 mL of glacial acetic acid and 4.5 g of sodium chlorite were added to the reactants again. This process was repeated a total of six times, with each repetition occurring every 1 h to ensure a uniform mixture of CSP and the chemicals. The resulting mixture was filtered and washed repeatedly with distilled water until reaching a neutral pH (approximately 7). The obtained holocellulose was then oven-dried for 48 h, and the yield of holocellulose was calculated based on the initial mass of CSP and the amount of holocellulose.

A total of 10 g of holocellulose was placed into a 250 mL aqueous solution of 17.5% NaOH and reacted at 20 °C for 40 min. Then, an additional 250 mL of distilled water was added. After 5 min, the mixture was filtered, and the resulting filtrate was soaked in 400 mL of 10% acetic acid solution. The cellulose was washed three times with 100 mL of boiling water and filtered. The cellulose was then placed in a vacuum drying oven and dried at 105 °C for 48 h. The cellulose incorporated into the PLA/PBS films underwent a sieving process using a 300 mesh screen prior to its utilization.

The holocellulose and cellulose yield was expressed as a percentage of CSP using (Equation (1)).

(1) $$Yield\;(\%)=\frac{M_{f}}{M_{i}}\times 100$$

where M_i_ is the initial weight of CSP and M_f_ is the final dried weight of holocellulose or extracted cellulose.

The sample for hemicellulose removal was prepared as follows: 10 g of CSP was placed in 250 mL of a 17.5% NaOH aqueous solution. The mixture was allowed to react at 20 °C for 40 min, and then 250 mL of distilled water was added. After a 5 min interval, the resulting mixture was filtered and the collected material was transferred to 400 mL of a 10% acetic acid solution. Thorough stirring was performed for 10 min to ensure complete neutralization of both NaOH and acetic acid. To eliminate any remaining impurities, the obtained sample was repeatedly rinsed with boiling water until reaching a neutral pH.

### 2.3. Preparation of PLA/PBS/Cellulose Blended Films

The solvent casting method was employed to prepare PLA/PBS films as follows. A mixture of 0.45 g PLA pellets and 0.05 g PBS pellets (totaling 0.5 g) was dissolved in 25 mL of DCM and stirred for 4 h at room temperature. The resulting solution was then subjected to ultrasonic dispersion and cast onto a glass petri dish for evaporation under ambient atmospheric conditions. To enhance the thermal properties of the PLA/PBS films, four different concentrations of cellulose (0.3, 0.5, 1, and 3 wt.% of PLA/PBS) were incorporated into the films. The thicknesses of PLA/PBS composite films were 30 ± 2 μm.

### 2.4. Characterization of Primary Components of Coconut Shell and Composite Films

The morphology of CSP, holocellulose, and extracted cellulose was examined with an optical microscope (CX31, OLYMPUS, Tokyo, Japan).

X-ray diffraction (XRD) was used to examine the crystallinity of CSP, cellulose, holocellulose, and hemicellulose extracted samples. The samples were analyzed in an X-ray diffractometer (Model: Rigaku D/max 2200, Tokyo, Japan) using Cu-Kα radiation (λ = 0.154 nm) at 40 kV and 30 mA with a goniometer speed of 0.02 s^−1^. The spectra were measured for 2θ in the range of 5–60°. The X-ray detector used was a scintillation counter with a detector angle of 40°, placed at a distance of 300 mm. The crystallinity index was calculated using Equation (2) as suggested by Segal, Creely, Martin Jr., and Conrad (1959) [39].

(2) $$Crystallinity\;index=\frac{I_{002}-I_{am}}{I_{002}}\times 100$$

where I_002_ is the peak intensity of the 002 lattice diffraction at 2θ = 22.8° (crystalline regions) and I_am_ is the intensity of the diffraction at 2θ = 18° considering the amorphous region.

The primary components of coconut shells were analyzed in an FT-IR spectrometer (ThermoFisher IS 10, Massachusetts, America) in reflection mode with the ATR accessory (resolution 4 cm^−1^, range 4000 to 650 cm^−1^, 64 scans).

Thermal weight loss analysis was conducted using a thermogravimetric instrument (TGA 2, Mettler Toledo, Zurich, Switzerland). In this analysis, approximately 8–10 mg of the polymeric materials were placed in an aluminum dish under a nitrogen (N_2_) atmosphere at room temperature. The sample was then subjected to heating at a rate of 10 °C/min using a high-resolution dynamic mode until reaching a temperature of 600 °C. Throughout the heating process, the weight loss of the sample in response to the temperature change was recorded.

The morphology of cryofractured cross sections of the PLA/PBS composite films containing cellulose was examined with a field emission scanning electron microscope (SEM) (HITACHI S4800, Tokyo, Japan) operating at 5 kV. The samples were fractured in liquid nitrogen and sputter-coated with gold. 

The crystallizing effect of cellulose on the composite films was evaluated using differential scanning calorimetry (DSC 3, Mettler Toledo, Zurich, Switzerland) on around 2–4 mg of the sample at a heating/cooling rate of 10 °C/min from 25–190 °C in an aluminum pan, under a nitrogen flow of 50 mL/min. The crystallinity degree (X_c_) was calculated with Equation (3):

(3) $$X_{c}(\%)=(\frac{{∆H}_{m}-{∆H}_{cc}}{{∆H}_{0}*\frac{1-wt.\%\left(cellulose\right)}{100}})\times 100$$

where ∆H_m_, ∆H_cc_ and ∆H_0_ are the experimental melting enthalpy, cold crystallization enthalpy, and the theoretical heat of fusion of 100% crystalline PLA (∆H_0_ = 93 J/g) and PBS (ΔH_0_ = 110.3 J/g), respectively [12,40].

The mechanical properties of the films (1.5 cm × 9 cm) were evaluated with a universal testing machine (GBH 2, GBPI, Guangzhou, China) at room temperature using a cross-head speed of 50 mm/min and a tensile load of 0.5 kN, according to ISO 527-3: 2018.

The light transmittance of the films was evaluated using a UV-Vis spectrophotometer (UV 5200PC, Shimadzu, Kyoto, Japan) in the range of wavelength from 800 nm to 200 nm. 

## 3. Results and Discussion

### 3.1. Morphology and Yield of Cellulose 

**Figure 1:**
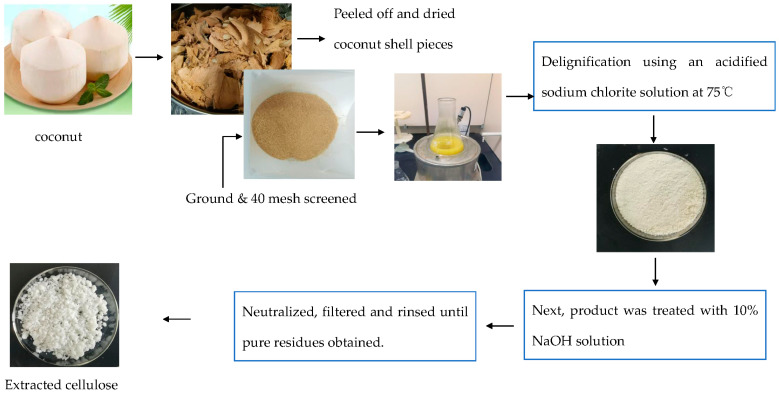
Schematic of extraction of cellulose using coconut shells.

**Figure 2:**
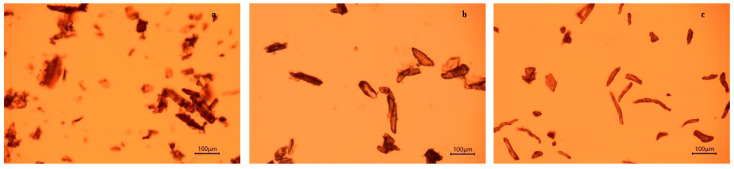
Optical microscopic images of (**a**) coconut shell powder, (**b**) holocellulose, and (**c**) cellulose.

White coconut shells were utilized as the raw materials to mitigate the impact of chromophoric groups on the purity and yield of cellulose (Figure 1). After grinding and drying, the coconut shells underwent oxidation, resulting in a light brown color. The color change from brown to off-white after each successive treatment confirmed the progressive removal of hemicellulose, lignin, and lipid and the subsequent increase in cellulose content of the CSP. Optical microscopic images clearly demonstrated that delignification did not significantly alter the diameter and length of CSP but imparted a smoother surface to the holocellulose (Figure 2). The final cellulose yield, after delignification and alkali treatment, was approximately 32.8% of the initial weight of CSP. These findings were consistent with the cellulose yield reported by Kalla [33]. The diameter and length of cellulose decreased compared to the initial CSP due to fibrillation. Similar reductions in cellulose diameter were previously observed in studies involving cellulose extraction from coffee and rice husks [41].

### 3.2. Crystallinity of CSP and Primary Components

**Figure 3:**
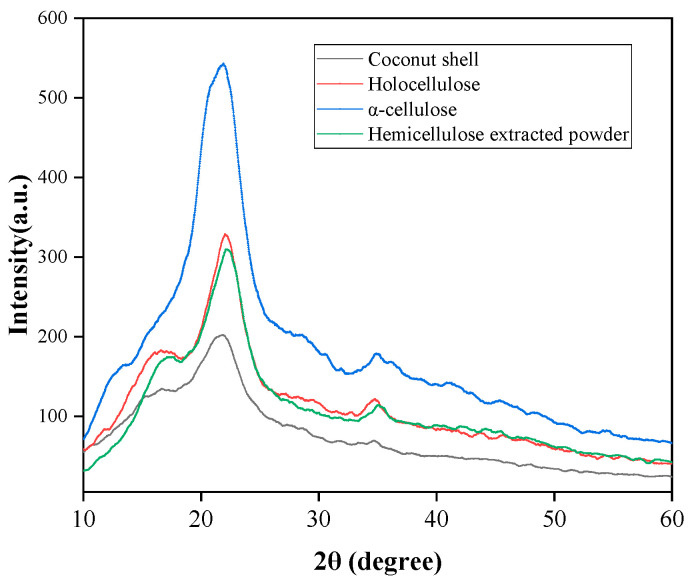
X-ray diffraction patterns CSP, holocellulose, cellulose, and hemicellulose extracted powder.

The X-ray diffraction patterns of CSP, holocellulose, cellulose, and hemicellulose extracted are shown in Figure 3. The patterns were typical of semicrystalline cellulosic materials with an amorphous broadband and distinct crystalline peaks. In all the samples analyzed, the diffractogram profile indicated the prevalence of cellulose type I (JCPDS 50-2241), with the primary intensity observed at about 2θ = 22.2° (plane 002) and identifiable peaks at 2θ = 17.1° (plane 101) and 34° (plane 004) [35]. The delignification and alkali treatment applied to CSP resulted in an enhanced crystallinity of the fibers, primarily due to the dissolution of amorphous compounds, including hemicelluloses and lignin. Consequently, the chemically treated samples exhibited a crystalline structure consisting of polymorphs of cellulose type I, wherein the cellulose molecules are aligned in parallel. The crystallinity of the extracted samples, including holocellulose, cellulose, and hemicellulose, increased to 47%, 54.3%, and 45.7%, respectively, in comparison to the original CSP (32.7%). These findings suggest that the proportion of the crystalline fraction increases as the amorphous constituents are removed.

### 3.3. Spectroscopic Characterization

**Figure 4:**
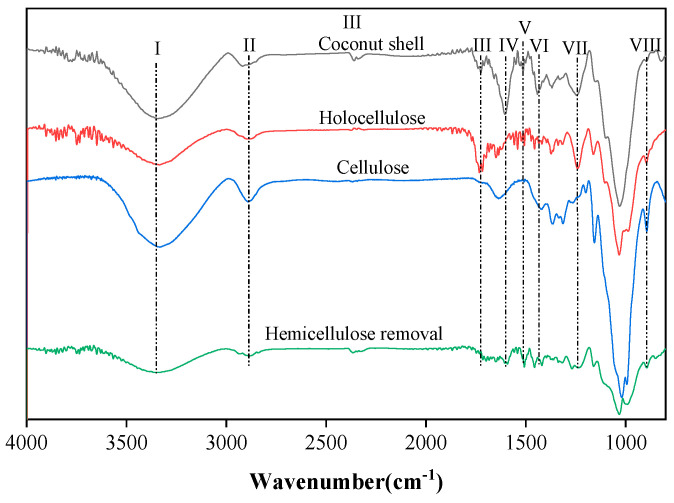
Fourier transform infrared spectra (800 to 4000 cm^−1^ wavenumber) of CSP, holocellulose, cellulose, and hemicellulose extracted powder, (I) 3340 cm^−1^, (II) 2890 cm^−1^, (III) 1735 cm^−1^, (IV) 1608 cm^−1^, (V) 1511 cm^−1^, (VI) 1458 cm^−1^, (VII) 1247 cm^−1^, (VIII) 895 cm^−1^.

The FTIR spectra of CSP, CSP, holocellulose, cellulose, and hemicellulose extracted powder are presented in Figure 4. The bands at 1458 (VI) and 1247 (VII) cm^−1^ were attributed to the bending of CH_3_ groups and the out-of-plane stretching vibrations of C=O in aryl groups. The peaks at 1608 (IV) and 1511 cm^−1^ (V) corresponded to the stretching vibrations of the C=C bonds in the aromatic ring of lignin [42]. These peaks completely disappeared in the extracted cellulose after chemical treatment, indicating the successful removal of lignin from the CSP fibers. The band observed at 1735 cm^−1^ (III) in CSP can be ascribed to the stretching vibration of C=O in acetyl and uronic ester groups of hemicellulose. This band was absent in cellulose and hemicellulose removal samples, suggesting the effective removal of hemicellulose through mercerization. The broad bands centered at 3340 cm^−1^ (I) and 2890 cm^−1^ (II) were associated with the stretching vibration of OH groups, which are observed in hydrophilic materials [43]. The peak observed at 2890 cm^−1^ in cellulose was relatively more intense, indicating a higher number of OH groups in cellulose. The most prominent peak, centered at 895 cm^−1^ (VIII), was assigned to the β-(1→4) linked glycosidic bonds in cellulose [34]. This peak became sharper after treatments, particularly in the case of cellulose, which experienced a substantial increase in cellulose content. In summary, characteristics observed in the FTIR spectrum confirm the successful preparation of cellulose from coconut shells.

### 3.4. Thermal Analysis of Fibers

**Figure 5:**
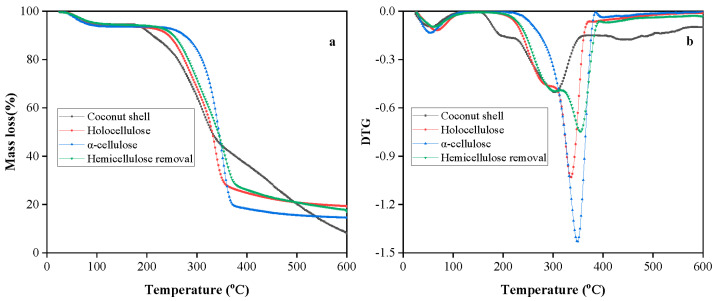
TGA (**a**) and DTG (**b**) curves of CSP, holocellulose, cellulose, and hemicellulose removal powder.

Figure 5a,b displays the thermogravimetric analysis (TGA) and the corresponding derivative (DTG) curves of analyzed samples, respectively. All the samples exhibited the first decompose intensity at 55 °C, mass loss of 2.5%, corresponding to the desorption of physically and chemically bound water and volatiles. Hemicellulose is made of short, branched heteropolysaccharides that are built up from various C5 (pentoses, such as xylose and arabinose) and C6 (hexoses, such as glucose, galactose, and mannose) sugars with some amount of uronic acid. The above components of hemicellulose have a lack of crystallinity, causing low thermal stability in addition to a low degree of polymerization [44,45,46]. The decomposition of CSP started at 200 °C due to the presence of hemicellulose. The stable cellulose decomposed at around 300 °C and exhibited different thermal stability than hemicellulose due to inherent differences in chemical structure, even though both are polysaccharides. CSP, holocellulose, and hemicellulose removal particles dropped significantly, accounting for subtracting the amount of hemicellulose and lignin pyrolyzed within this temperature range. Cellulose decomposed in a rather narrow range compared to CSP, and the maximum pyrolysis rate of pure cellulose was at 347.3 °C, and 18% of residues were retained at about 400 °C. For the treated samples, the removal of lignin or hemicelluloses generated higher solid residues such as ashes and inorganic materials (around 20% of the initial mass). The third additional event (not observed for raw ground materials) occurred from 420 to 500 °C, with a maximum at 440 °C of CSP. It was ascribed solely to the degradation of lignin (the most stable of the three compounds). Above this temperature, pyrolysis occurred, which was associated with the release of carbon dioxide and consequent reduction in the residual mass. Cellulose and hemicellulose exhibit different thermal stability due to inherent differences in chemical structure, even though they are polysaccharides.

### 3.5. Morphology of Films

**Figure 6:**
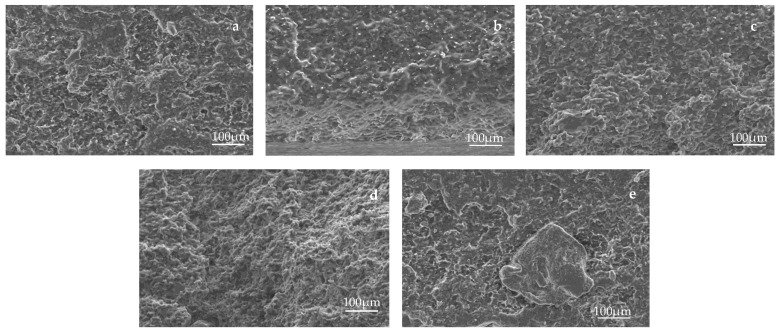
SEM images (**a**–**e**) of cryofractured surface of PLA/PBS composite films containing various cellulose content (0, 0.3, 0.5, 1, 3 wt.%).

Figure 6a–e shows the SEM images of the cryofractured surface of neat PLA/PBS films and PLA/PBS films containing 0.3, 0.5, 1, and 3 wt.%, respectively. It can be seen in Figure 6a that the neat PLA/PBS film exhibited an irregular and tough fracture pattern with ductile characteristics. PBS acted as a toughening agent in PLA/PBS films, imparted toughness, and resulted in a ductile fracture behavior of composite films. Similar morphology was reported in the literature when PLA was plasticized with PPG and natural rubber [47,48]. The irregular and non-linear crack propagation and deformation observed on the cryofractured surface of PLA/PBS films indicated that the addition of cellulose did not have a negative impact on the ductile fracture behavior of the films. However, agglomeration of cellulose occurred at the highest cellulose content of 3 wt.%, indicating poor dispersion in PLA/PBS films.

### 3.6. Effect of Cellulose on Crystallization Behaviour of Films

**Figure 7:**
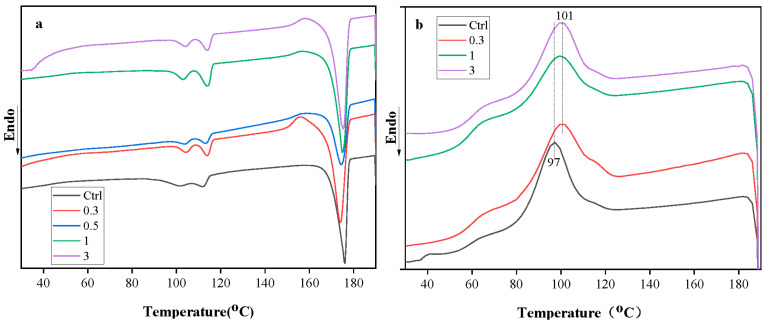
DSC thermograms of PLA/PBS films containing various cellulose content (0.3, 0.5, 1, and 3 wt.%) heating scan (**a**), cooling scan (**b**).

Figure 7 shows the DSC thermograms of PLA/PBS films containing various cellulose content, and Table 1. summarizes melting enthalpy (∆H_m_), crystallizing enthalpy (∆H_cc_), and crystallinity of PLA/PBS composite films containing different cellulose content. Melting temperatures of PLA at 175 °C were observed in all the analyzed samples during the heating scan; PBS adopted in this study exhibited two melting temperatures at 101 and 112 °C, respectively, mainly due to the raw PBS materials still containing some portion of PBSA. For a series of PLA/PBS blends at various cellulose contents, the crystallization temperature of PLA shifted to 101 °C and higher than 97 °C of neat PLA/PBS (Figure 7b). As the crystallization temperature represents the crystalline phase, the higher crystallization temperature signifies that cellulose enables the crystallization to begin at an early stage; this reflects the cellulose as a heterogeneous nucleating agent to enhance the crystal formation of the PLA/PBS blends. However, the addition of 0.3% and 0.5% cellulose content reduced the crystallinity of films compared to the neat PLA/PBS blends, while exceeding 1 wt.% cellulose content slightly increased the crystallinity of PLA/PBS films. The addition of a small amount of cellulose may disrupt the polymer’s molecular chain regularity and inhibit the folding mobility of crystallizable chain segments [49]. The crystallization temperature of PBS and PBSA shows 65 °C as a close shoulder of the crystallization of PLA. This suggests that the vibration of the cellulose chain during thermal treatment might effectively induce the movement of PLA/PBS matrices as both chains are somewhat miscible, which is in accordance with the results of the previous study. It reveals that cellulose acts as a heterogeneous nucleating agent and contributes to ununiformed crystallites varying in size, such that the crystallization peak of PLA/PBS/cellulose films at higher temperatures was formed on the cooling exothermic of the incomplete crystallization. Meanwhile, a small amount (0.3–0.5 wt.%) of cellulose took a dominant disruption role in PLA/PBS films, increased steric obstacles for segmental motion, and increased the rigidity of PLA/PBS chains. The preferable formation of crystalline regions with the increasing amount of cellulose (1, 3 wt.%) is mainly due to an increased interface interaction, via hydrogen bonding, between abundant hydroxyl groups of cellulose and polymers [37].

### 3.7. Effect of Cellulose on Thermal Stability of Films

**Figure 8:**
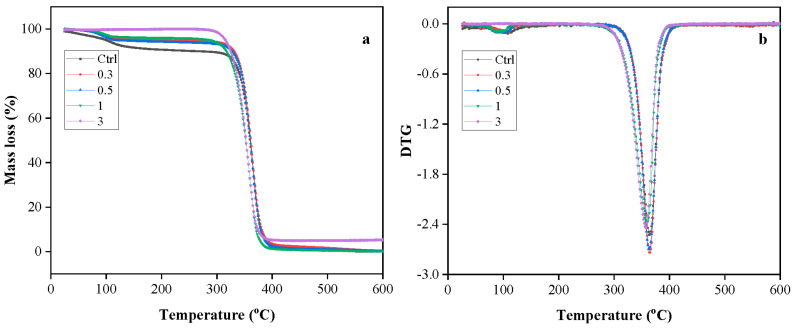
TGA (**a**) and DTG (**b**) curves of PLA/PBS films containing various cellulose content (0.3, 0.5, 1, and 3 wt.%).

Thermogravimetric analysis (TGA) was performed to study the effect of extracted cellulose on the thermal degradation behavior of PLA/PBS in the inert atmosphere. Figure 8a,b illustrate the TGA and derivative thermogravimetric (DTG) curves of the PLA/PBS films with varying cellulose content (0.3, 0.5, 1, and 3 wt.%). Table 2 summarizes the characteristic decomposition temperature (T_onset_ and T_max_) and residues at 600 °C of PLA/PBS films containing different cellulose content. It can be seen from Figure 8a that single-step degradation occurred for all the samples, with more than 90% mass loss occurring between 300 and 400 °C. As shown in Figure 8a and reported in Table 2, the presence of cellulose higher than 1 wt.% in the PLA/PBS decreased the onset decomposition temperature and maximum decomposition temperature of films. Acid hydrolysis can remove the amorphous region, producing a highly crystalline cellulose. Nevertheless, the different treatment variables and cellulose sources directly affect the degree of cellulose crystallinity [50]. Based on the crystallinity index of extracted cellulose (54.3%), it can be confirmed that extracted cellulose contained significant amorphous regions, which were less thermally stable than crystalline regions. Consequently, the thermal stability of films decreased as cellulose content increased to 1 wt.%. Furthermore, it is noteworthy that the films loading 3 wt.% of cellulose had a higher residual mass (5.47%) compared to neat PLA/PBS films (0.13%). Cellulose extracted from CSP is an organic material and even less thermostable than the PLA/PBS polymer matrix; the incorporation of cellulose reduced the burning rate by promoting more dehydration and char formation [51]. This finding suggests that the presence of cellulose contributes to the formation of more residual mass, potentially enhancing the flame resistance properties of the composite films.

### 3.8. Effect of Cellulose on Mechanical Property of Films

**Figure 9:**
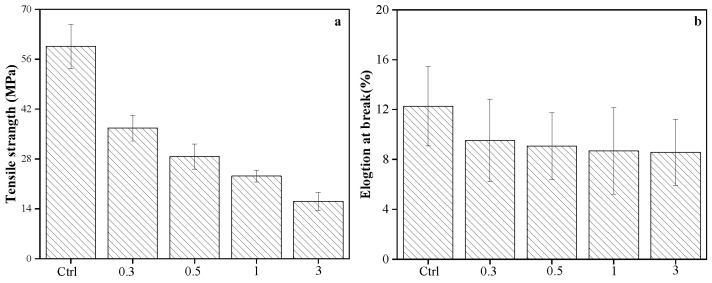
Tensile strength (**a**) and elongation at break (**b**) of PLA/PBS films containing various cellulose content (0.3, 0.5, 1, and 3 wt.%).

The effect of cellulose on the tensile strength and elongation at break of PLA/PBS/cellulose films is shown in Figure 9. Tensile strength and elongation at break of the PLA/PBS film were 59.6 MPa and 12.3%, respectively. Tensile strength was expressively decreased when increasing the content of cellulose, from 59.6 MPa for PLA/PBS blend to 36.6 MPa PLA/PBS composite having 0.3 wt.% of cellulose. Tensile strength for 3 wt.% of cellulose PLA/PBS composite film decreased to 16.2 MPa, which is equivalent to the tensile strength of PBAT/PLA films. The elongation at break of PLA/PBS film was 12.27%, whereas it slightly decreased to 8.57% for PLA/PBS composite films containing 3 wt.% cellulose. The decrease in mechanical property after the addition of cellulose was presumably due to the rigidity of cellulose fibers, weak interfacial affinity, and stress transfer property between cellulose and polymers. In addition, poor dispersion of 3 wt.% cellulose in PLA/PBS films further compromised structural integrity and decreased the overall strength of the films, which is consistent with the cyrofractured morphology of films. The results of film properties were in accordance with those of cellulose nanocrystal-reinforced PLA composites [52].

### 3.9. Effect of Cellulose on UV Transmittance of Films

**Figure 10:**
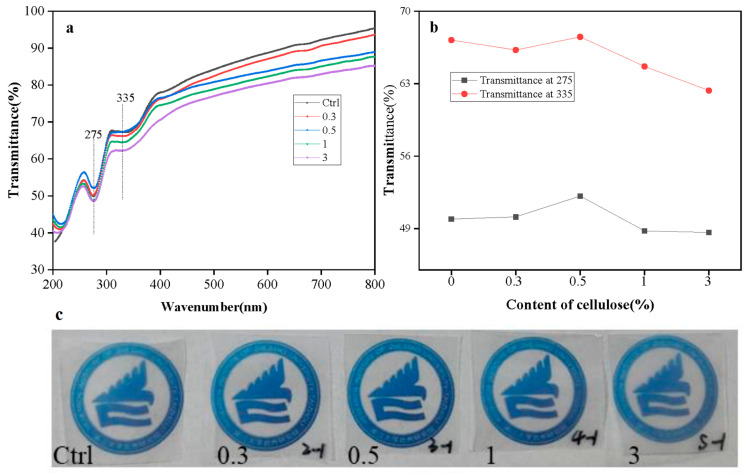
UV-Vis transmittance of the PLA/PBS composite films loading various cellulose content (0.3, 0.5, 1, and 3 wt.%) (**a**), transmittance of films at 275 nm and 335 nm (**b**), photographic images of PLA/PBS composite films (**c**).

Transmittance and camera images of a series of PLA/PBS films containing cellulose are shown in Figure 10a,c, and specific transmittance of films at 275 nm (UVC region) and 335 nm (UVA region) are summarized in Figure 10b. As can be seen, PLA/PBS film appears as a transparent matrix, and the PLA/PBS film maintains its transparency, even after the incorporation of 3 wt.% cellulose (Figure 10c). The transparency of films containing cellulose can be attributed to the miscibility between PLA and PBS and the limited influence of cellulose on the crystallinity of PLA/PBS films. The transmittance of films in the visible region (420–800 nm) decreased as the content of cellulose increased, and PLA/PBS films exhibited higher transmittance than the PLA/PBS films containing different amounts of cellulose. This may be attributed to the large grain size of cellulose that hindered the transmittance of visible light. Despite the reduction in transmittance in the visible region with an increase in cellulose content, films still had certain transparency. Regarding the transmittance values at 275 nm and 335 nm, PLA/PBS film with cellulose at 3 wt.% exhibited decreasing trends of 3% and 7.5%, respectively, compared to neat PLA/PBS films. This suggests that the incorporation of cellulose has the potential to enhance the UV barrier property of PLA/PBS films while leading to a slight loss of transmittance in the visible region (10%). There are still challenges in balancing the transmittance of PLA/PBS films with the full UV barrier potential of cellulose.

## 4. Conclusions

In summary, delignication and mercerization treatment processes were conducted in this work as a rapid, effective (with yields of more than 32%), and eco-friendly approach for the extraction of cellulose from coconut shells. The optical images of extracted cellulose showed a reduction in fiber diameter as compared to CSP because the composite fibril structure was broken into individual cellulose micro-fibrils after the removal of lignin and hemicellulose. The absence of lignin and hemicellulose in extracted cellulose was confirmed from FTIR spectra. The extracted cellulose had a crystallinity of 54.3% and had better thermostability as compared to raw CSP.

In addition, the extracted cellulose was applied to reinforce solvent-casting PLA/PBS films; uniform distribution of cellulose in PLA/PBS films can be achieved at the highest concentration of 1 wt.%, and all the composite films showed excellent transparency even when 3 wt.% of cellulose was added. The thermal stability of the PLA/PBS composites containing cellulose (>1 wt.%) was lower than the PLA/PBS films. However, the incorporation of 3 wt.% cellulose reduced the burning rate of films by promoting more char formation. Cellulose acted as a heterogenous nucleation agent to promote the formation of non-uniform crystals, while cellulose had a slightly negative impact on crystallinity due to the disruption of polymer chains at lower cellulose content (0.3, 0.5 wt.%). This finding suggests that the presence of cellulose contributes to the formation of more residual mass, potentially enhancing the flame resistance properties of the composite films. The incorporation of cellulose as reinforcing fibers in PLA/PBS films decreased the mechanical properties of the composite films considerably. Moreover, the film incorporated with 3 wt.% of cellulose exhibited promising UV barrier properties, as the transmittance slightly decreased in the UVA and UVC regions compared to PLA/PBS films. Despite the reduction in transmittance in the visible region with an increase in cellulose content, films still had certain transparency. The present study may help to solve the environmental hazard issue regarding the solid waste of packaging with commodity plastics by considering replacement with biodegradable PLA and PBS polymer blends and functional composites.

## Figures and Tables

**Table 1:** Melting enthalpy (∆H_m_) of PLA and PBS, crystallizing enthalpy (∆H_cc_), and crystallinity (X_c_) of PLA/PBS in films containing different contents of cellulose.

Formulation	∆H_mPBS_ (J/g)	∆H_mPLA_ (J/g)	∆H_cc_ (J/g)	Xc (%)
Ctrl	4.4	37.42	15.77	27.34
0.3	5.38	37.49	23.53	20.36
0.5	3.46	26.63	11.19	19.93
1	8.47	38.75	19.58	29.34
3	5.61	38.7	20.14	29.12

**Table 2:** Characteristic decomposition temperature (T_onset_ and T_max_) and residues at 600 °C of PLA/PBS films containing different cellulose content.

Formulation	T_onset_ (°C)	T_max_ (°C)	Residue (%)
Ctrl	339.6	363.6	0.13
0.3	341	365	0.05
0.5	339.6	363.6	0
1	329	359.6	0.15
3	327.6	357	5.47

## Data Availability

The data used to support the findings of this study are available from the first author upon request.
